# Repair of total anomalous pulmonary venous return (TAPVR) with pulmonary artery (PA) Stenosis in adult

**DOI:** 10.1186/1749-8090-10-S1-A70

**Published:** 2015-12-16

**Authors:** Fuad Z Abdullayev, Imadaddin M Bagirov, Nigar J Kazimzade, Larisa S Shikhiyeva

**Affiliations:** 1 Dept. Cardiac Surgery, Topchibashev Research Centre of Surgery, Baku, AZ 1122, Azerbaijan

## Background/Introduction

Less than 7% patients with TAPVR survive into adulthood. Predictors of survive in untreated patients are: absence of concomitant CHD & atrial isomerism; large ASD; low pulmonary vascular resistance; mild/moderate TV incompetence. Experience of TAPVR repair in adults restricted by prevalence of case reports. Majority of repaired were ≥50 years old. There have been four case reports of untreated TAPVC diagnosed after 60 years of age, and three of them underwent successful surgery on 61[J Melki,1992], 63 [MH McMullan,1992] and 66 years of age[U Wetzel,2010]. Large surgical series presented by X Jian, 2012 [12 pts], S Talwar, 2008[27 pts], B Korbmacher, 2001[12 pts] and J Rodriguez-Collado, 1992 [19 pts]. Among case reports of TAPVR in adults, up to our knowledge, there are only 4 patients with TAPVR and with PA valve stenosis /RVOT obstruction.

## Aims/Objectives

To present successful repair of TAPVR with severe PA stenosis in 22 years old female

## Method

A 22-year-old female was referred to our unit for the cyanotic CHD. She had been diagnosed for CHD at the age of 9 years. Worsening on last 3 years. Body Mass 50 kg. Fatigue, cyanosis, markedly clubbed fingers, "watch-glass" nails. SaO2= 68-72%. Sinus rhythm. Echo- and 3D CT-scan revealed: Situs Solitus, normal heart position. TAPVR Darling's IV (supracardiac-cardiac), large ASD II, severe PA valve stenosis with SGr across the valve 119 mm. Hg. LV EDD-32 mm, LV ESD-26 mm. NYHA III.

## Results

09/04/2015 - Repair Of TAPVR ("Double Patch" technique) and PA Valvuloplasty. SpGr across PA valve decreased to 31 mm.Hg. The patient made an uneventful recovery and discharged from the hospital on 9th day after surgery. 5 months later surgery: patient was without any complaints, free from MACCE, SaO2 = 98 - 99%. SGr across PA valve 30 mm Hg.

## Discussion/Conclusion

1.TAPVR in adults could be repaired with 0% mortality; 2. Determinants of long-term events-free survival: pulmonary vascular resistance ≤4-6 u/Wood; lack of post/op PV obstruction; mild/moderate TV incompetence; sinus rhythm

## Consent

Written informed consent was obtained from the patient for publication of this abstract and any accompanying images. A copy of the written consent is available for review by the Editor of this journal.

